# Rest assured: Dynamic functional connectivity and the baseline state of the human brain

**DOI:** 10.1162/imag_a_00365

**Published:** 2024-11-19

**Authors:** Enzo Tagliazucchi

**Affiliations:** Institute of Applied and Interdisciplinary Physics (CONICET) and Department of Physics, University of Buenos Aires, Buenos Aires, Argentina; Latin American Brain Health (BrainLat), Universidad Adolfo Ibáñez, Santiago, Chile

**Keywords:** resting state, dynamics, cognition, functional connectivity, complex systems, brain states

## Abstract

While dynamic functional connectivity remains controversial in human neuroimaging, the transient nature of interareal coupling is considered a robust finding in other fields of neuroscience. Nevertheless, the origin and interpretation of these dynamics are still under debate. This letter argues that ongoing cognition is not sufficient to account for dynamic functional connectivity. Instead, it is proposed that the baseline state of the brain is inherently unstable, leading to dynamics that are of neural origin but not directly implicated in cognition. This perspective also reinforces the usefulness of conducting experiments during the resting state.

The advent of modern neuroimaging techniques in the late 1980s and 1990s contributed dramatically to our knowledge of the mapping between function and structure in the brain. Methods such as positron emission tomography (PET) and functional magnetic resonance imaging (fMRI), in combination with task-based paradigms, were applied to the systematic investigation of the neural correlates of behavior and cognition (Raichle, 2001). Older techniques, such as electroencephalography (EEG), were empowered by increased computational power and algorithmic sophistication (Michel & Murray, 2012). Neuroimaging has also been applied to investigate the spontaneous or unconstrained fluctuations of brain activity, that is, activity measured in the absence of instructions besides resting with eyes closed while avoiding sleep (Biswal, 2012;Snyder & Raichle, 2012). The study of resting-state activity eventually demonstrated that spontaneous activity shows a complex and reproducible organization, which is not only modulated by ongoing behavior and cognition in healthy individuals, but can also be disrupted in idiosyncratic ways in specific neuropsychiatric conditions (Greicius, 2008;Van Den Heuvel & Pol, 2010).

An important factor behind the surge of resting-state studies over the last decades is the introduction of functional connectivity as a tool to analyze brain activity (Biswal et al., 1995). Two regions of the brain are functionally connected if they show some degree of covariance over time, usually estimated using linear correlations, but sometimes with more sophisticated methods (Bastos & Schoeffelen, 2016). The concept of functional connectivity is complementary to that of structural or anatomical connectivity, which refers to two regions being linked by white matter fiber tracts. In recent years, resting-state studies increasingly began to focus on dynamic functional connectivity, that is, the computation of functional connectivity over short periods of time to infer the transient coupling and uncoupling of brain regions (Hutchison et al., 2013).

The critical assessment of resting-state studies has stirred several controversies in the field. Perhaps the most obvious issue concerns the nature of resting-state activity fluctuations and their relationship with cognition and information processing in the brain. Initially, criticism focused heavily on identifying potential non-neural contributions to the spontaneous signals measured using fMRI, which include head motion (Power et al., 2014), artifacts of cardiac or respiratory origin (Birn, 2012), fluctuating vigilance, and even the onset of deep sleep during the measurements (Tagliazucchi & Laufs, 2014). However, multimodal studies combining fMRI with electrophysiological recordings contributed to mitigate these concerns (Schölvinck et al., 2010;Tagliazucchi & Laufs, 2015). The interpretation of dynamic functional connectivity analyses has also been questioned, as fluctuations in functional connectivity can also emerge from physiological confounds, motion artifacts, fluctuating vigilance, and other non-neural factors (Lurie et al., 2020). Moreover, the use of certain methods to compute changes in functional connectivity (e.g., correlations computed over sliding windows) without the appropriate implementation of null models may give the false impression that connectivity is time-dependent, when, in fact, fluctuations are to be expected even when coupling is stationary (Zalesky & Breakspear, 2015). This potential methodological limitation was overturned by the application of more sophisticated models for the inference of functional connectivity dynamics, such as Hidden Markov Models (HMM) (Vidaurre et al., 2017).

In my view, while some of these objections are still worthy of research, considerable evidence suggests that functional connectivity changes over time and that these changes, indeed, originate from neural sources. Multimodal neuroimaging studies have established that functional connectivity fluctuations are correlated with concurrent changes in neuroelectric activity as indexed by local EEG spectral power, by combining EEG and fMRI in awake humans with eyes closed (Chang et al., 2013;Keilholz, 2014;Tagliazucchi et al., 2012;Tagliazucchi & Laufs, 2015). Also, the transient nature of interareal synchronization is well supported by studies conducted in animals, some of them addressing temporal scales similar to those measured with fMRI (Pais-Roldán et al., 2021;Schölvinck et al., 2013;Vinck et al., 2013). Examples can be found in the studies byWang et al. (2012)andShi et al. (2019), showing that slow oscillations (<20 Hz) of local field potentials (LFP) correlate with fMRI functional connectivity fluctuations in anesthetized macaque and squirrel monkeys, respectively. Many of the objections targeted to the indirect nature of fMRI recordings do not apply to similar results obtained using EEG and magnetoencephalography (MEG), also revealing functional connectivity dynamics related to those seen in fMRI (Hipp and Siegel, 2015). Dynamic functional connectivity is known to covariate with cognitive processing, behavior, and individual traits (Cohen, 2018;Liégeois et al., 2019;Vidaurre et al., 2021). However, some deep issues persist concerning the interpretation of dynamic functional connectivity; in particular, concerning the role that ongoing cognitive processing plays in the origin of these dynamics (Lurie et al., 2020).

In recent years, different positions have been defended regarding the role of cognition in the dynamics of brain activity and connectivity during rest. On the one hand, it has been argued that these fluctuations are optimally understood as an aggregation of neural activity sources related to well-defined but unknown ongoing cognitive processes (Cohen, 2018). Conversely, others have proposed that fluctuations in brain activity and connectivity may emerge from sources unrelated to cognition, such as physiological, metabolic, and homeostatic processes (Laumann & Snyder, 2021). In the following, I defend another possibility: the dynamic nature of brain activity and connectivity is best understood as the consequence of how neurons behave and are interconnected in the brain, so that the explanation for these phenomena should be, at least in part, formulated within the framework of dynamical systems theory. This idea has been pioneered over the last two decades by a combination of empirical neuroimaging research, theoretical work, and computational modeling (Breakspear, et al., 2010;Breakspear, 2017;Cabral et al., 2017;Chialvo, 2010;Deco et al., 2009;Ghosh et al., 2008;Griffiths et al., 2021;Honey et al., 2007;Markram, 2012). In particular, due to the structural and neurochemical properties of neural networks, their collective behavior is inherently unstable, thus contributing to the transient coupling between brain regions and to the emergence of a dynamic baseline state, that is, a state of rest that is constantly active and influences ongoing cognitive processing. This is supported by evidence coming from multiple empirical and modeling studies showing that complexly structured brain activity remains during states of diminished consciousness and cognition (Boly et al., 2008) and that resting-state activity accounts for the majority of the energy consumption of the brain (Raichle, 2006). Also, this dynamic baseline interacts with evoked activity, influencing ongoing cognition and sensory processing, and may also introduce spontaneous fluctuations in attention and arousal (Sadaghiani et al., 2010).

If the dynamics of brain activity and connectivity, indeed, reflect ongoing cognition, it could be argued that resting-state studies are always less informative than those based on paradigms that include sensory stimulation or task engagement (Finn, 2021). However, proposals to neglect the study of resting-state activity dynamics diverge from the multi-level approach that has been successful in other areas of biology. These proposals are predicated on a viewpoint that favors the explanatory power of cognitive science over other approaches toward understanding neural activity and its behavioral implications. According to this viewpoint, the main objective is to understand how the brain acts as an information processing device representing and transforming information with the objective of guiding behavior. At a coarse scale, the information processing functions of the brain are identified with facets of cognition such as perception, attention, different forms of memory, decision making, language, and others. Even when subjects are behaviorally in a state of rest, their spontaneous thoughts may rely on the coordination of these cognitive functions. Implicit in this viewpoint is a compartmentalization between cognitive function and its neurobiological substrate—or, in other words, between computation and the underlying dynamics supporting these computations. This compartmentalization owes much to the long-standing parallelism between minds/software and brains/computers (Brette, 2022). Yet, as I will discuss below, computation and dynamics in the brain may be more intertwined than could be expected from this analogy.

In contrast to human-engineered systems, the understanding of biological systems requires to integrate and relate multiple levels of explanation (Rivelli, 2019). Ideally, one could strive to understand neural networks solely in terms of their information processing capacities, for instance, by the input/output transformations they implement. However, these transformations are, in turn, limited by the dynamical repertoire of the interconnected neurons in the network (Vyas et al., 2020). Thus, unveiling the laws that govern the dynamics of neurons is a necessary step to understand how networks of neurons compute, as well as what they can compute. In turn, understanding these dynamics requires an approach that is focused on how the behavior of the units that comprise the system depends on their mutual interactions, typically described by computational neuroscientists using deterministic or stochastic differential equations (Deco et al., 2008). As shown by experiment, the processing of information in the brain seems to be linked to specific kinds of collective dynamics, including transient functional connectivity, but also synchronous oscillations (Ward, 2003), traveling and standing waves (Muller et al., 2018), and scale-free avalanches (Pasquale et al., 2008;Shew & Plenz, 2013), among other phenomena. In models of whole-brain activity, these dynamics arise as a consequence of noise-driven multistability (such as the noise-driven Hopf bifurcation between oscillatory and fixed-point dynamics) or deterministic chaos (Cofre et al., 2020;Deco et al., 2017;Piccinini et al., 2021). Moreover, the local dynamics of models successfully fitted to fMRI data can be reduced to the canonical form of a Hopf bifurcation (Piccinini et al., 2022). At the level of neural circuits, studying the spontaneous behavior of neurons is crucial to understanding their dynamics when subject to stimulation (Ringach, 2009). This is because the spontaneous activity of neural ensembles plays by itself a role in computation: it is a dynamic baseline, a state of continuous activity that facilitates the optimal processing of incoming signals (Deco et al., 2011).

Neuroimaging can be used to shed light on human cognition, but also on the large-scale functional connectivity dynamics of baseline activity. Severing afferent and efferent neural pathways is a traditional method of investigating the baseline activity of isolated brains; however, obvious ethical considerations limit these experimental manipulations to animal models. Although typical resting-state activity recordings are not obtained from isolated brains, their analysis, nevertheless, supports the consolidation and maintenance of a dynamic baseline. The complex spatial and temporal organization of fMRI resting-state functional connectivity is not only measured during conscious wakefulness but also in states where cognitive processing is considered to be generally impaired, such as under general anesthesia (Barttfeld et al., 2015), during deep sleep (Tagliazucchi & Laufs, 2014), or even in unconscious brain-injured patients (Demertzi et al., 2019). While resting-state activity is modified during these states, the extent of these alterations is minor when compared to the overall preservation of resting-state networks (Mashour & Hudetz, 2018), that is, group of regions presenting coordinated activity fluctuations during rest (Damoiseaux et al., 2006). Moreover, as in the case of isolated neural circuits, the processing of incoming information depends on the dynamics of large-scale baseline fluctuations in activity and connectivity, which can explain the behavioral inter-trial variability measured in certain tasks (Palva et al., 2013;Sadaghiani et al., 2010). Finally, the spatiotemporal patterns of activity measured during rest can be put into correspondence with task-evoked activity maps, suggesting that the repertoire of behaviorally relevant large-scale brain configurations is visited spontaneously over time (Smith et al., 2009). This is consistent with resting-state connectivity reflecting a baseline state of continuous activity that approximates the configurations associated with specific behavioral responses, thus being advantageous from an evolutionary perspective (Deco et al., 2011).

Even if the merits of studying the dynamics of resting-state activity are accepted, some important methodological disagreements may persist. For instance, the use of naturalistic sensory stimuli during fMRI recordings may reduce test-retest variability, yielding increased sensitivity to individual traits, behaviors, and group differences (Finn & Bandettini, 2021;Gal et al., 2022). Indeed, when seen as a complex dynamical system, it is reasonable to assume that perturbing the brain in a reproducible manner will reduce variance and increase the signal-to-noise ratio, as the repertoire of the states that are visited over time can be reduced by means of external stimulation and task engagement (Ponce-Alvarez et al., 2015). However, these practical considerations do not invalidate questions related to the exploration of the larger set of transient connectivity states occurring during rest. Why does this exploration occur? What is the repertoire of possible states? How is this repertoire determined by structural and/or neurochemical features of the brain, such as structural connectivity patterns or the action of specific neuromodulators? To make progress in these questions, amplifying individual variability may actually be a better strategy than attempting to minimize it. This applies especially to disorders where the unconstrained wandering of consciousness and cognition (or lack thereof) displays pathological characteristics, such as attention disorders, obsessive-compulsive disorder, and ruminative thoughts in depression.

Indeed, the study of certain temporally extended brain states could be optimal during rest. Typical examples include mind-wandering (Kucyi & Davis, 2014), as well as the onset and consolidation of sleep (Damaraju et al., 2020). Why does the mind eventually wander, and why does wakefulness eventually evolve into sleep? The deterministic nature of these processes suggests an explanation in terms of the dynamics of networked neurons and their functional connectivity, together with the effect of neuromodulatory systems (Arendt & Skene, 2005;Kringelbach et al., 2020;Shine et al., 2021). Sleep occurs locally in the cortex (Siclari & Tononi, 2017), and in organisms with brains that are dramatically less complex than the mammalian brain (Shaw et al., 2000). In both cases, its emergence can be tracked to the dynamical implications of processes occurring at the cellular scale (Pace-Schott & Hobson, 2002). Considerable advances have been made concerning neural dynamics during sleep (Amzica & Steriade, 2001;Nita et al., 2007;Sanchez-Vives, 2020;Steriade et al., 2001), the regulation of sleep by specific neural nuclei (Saper and Fuller, 2017), and the computational modeling of sleep-like activity based on realistic biophysical assumptions (Hill & Tononi, 2005). Even though cognition can occur during sleep (Goupil & Bekinschtein, 2012), it is a reasonable starting point to investigate the properties of the dynamic baseline. This is complemented by animal modeling studies showing that complex spontaneous activity and connectivity are not only linked to cognition, but also manifest under sleep and anesthesia in rats and mice studied with electrophysiology, fMRI, and ultrafast fMRI (Cabral et al., 2023;Grandjean et al., 2023;Gutierrez-Barragan et al, 2019). Mind-wandering could be a phenomenon similar to sleep, as it might originate from local sleep or sleep-like processes in specific regions of the cortex (Andrillon et al., 2021). These spontaneous but deterministic phenomena are best understood as dynamical consequences of the properties of neural networks, and the inclusion of sensory stimulation (or other externally induced manipulations) may actually obscure their study instead of facilitating it.

It has been argued that the brain is never truly at rest, but constantly engaged in spontaneous and potentially effortful cognition; thus, despite the instructions given by the experimenters, subjects never manage a true resting state devoid of thoughts and cognition. However, the brain is also never truly engaged in a task, since the focus of attention is labile and subjects easily drift away from external demands toward internal thoughts that are impossible to fully capture by the experimenters. Faced with this difficulty, an alternative is anchoring these fluctuating thoughts by means of naturalistic stimuli, or other paradigms that depart from a typical resting-state study. This is clearly a valuable strategy, as it has already shown promise in neuroimaging studies. However, a different but equally important objective is to understand why the brain is so inherently unstable, why both rest and task engagement are so difficult to maintain over time, and why the baseline activity of the brain is dynamic and ever-changing with unpredictable yet characteristic patterns. I propose that an answer to these questions will not come from understanding the brain in terms of its information processing capacities. Instead, it will be obtained by investigating the dynamical properties of the brain, complemented with computational modeling efforts, and eventually formulated using the language of dynamical systems.
